# Phage therapy: assessment of the efficacy of a bacteriophage isolated in the treatment of salmonellosis induced by *Salmonella enteritidis* in mice 

**Published:** 2017

**Authors:** Farhad Nikkhahi, Mohammad Mehdi Soltan Dallal, Mahmood Alimohammadi, Abbas Rahimi Foroushani, Zahra Rajabi, Fatemeh Fardsanei, Seyed Mostafa Imeni, Parisa Torabi Bonab

**Affiliations:** 1 *Division of Medical Bacteriology, Department of Pathobiology, School of Public Health (TUMS), Tehran, Iran.*; 2 *Food Microbiology Research Center, Tehran University of Medical Sciences (TUMS), Tehran, Iran.*; 3 *Environmental Health Engineering Department, School of Public Health, Tehran University of Medical Sciences, Tehran, Iran*; 4 *Department of Epidemiology and Biostatistics, School of Public Health, Tehran University of Medical Sciences, Tehran, Iran.*; 5 *BETA Technology Centre: “U Science Tech”, University of Vic- Central University of Catalonia, 08500 Vic, Barcelona, Spain*

**Keywords:** Bacteriophage, Salmonella enteritidis, Isolation

## Abstract

**Aim:**

This work aims to isolate and perform comparative studies of a phages active against a Salmonella enteritidis strain from Iran. Also, suitable phage candidates for therapy of mice will be selected.

**Background:**

Bacteriophage is of particular interest as a biocontrol agent in the prevention of food-borne illnesses. In recent years tend to use bacteriophages to control pathogenic bacteria has increased. A bacteriophage is considered to be a potent antibiotic alternative for treating bacterial infections.

**Methods:**

the specific phages against Salmonella Enteritidis was isolated and candidates for therapy of mice will be selected. Mouses divided into the six specific groups. Groups of mice were as follows: A: Bacteri (control) B: Bacteri+ bacteriophage (Simultaneous), C: Bacteri + bacteriophage Four days later, D: Bacteriophage + bacteri four days later E: Bacteri+ Ciprofloxacin (Simultaneous) F: Bacteri+ ciprofloxacin+ bacteriophage (Simultaneous).

**Results:**

In this study, a lytic bacteriophage is isolated and it shows that phage has a head size of 46 nm and without a tail, by using an electron microscope. Oral administration of a single dose of 2 × 109 PFU/mouse bacteriophage enable to protect mouse against salmonellosis and it causes treatment of salmonellosis in mice.

**Conclusion:**

The use of this phage compared to ciprofloxacin shows that in addition of the treatment of mouse, it also prevents weight loss.

## Introduction

 Salmonella enteritidis is a gram-negative bacterium and case Salmonellosis caused by ingestion of food contaminated with animal feces. In human salmonellosis mostly as occurs gastroenteritis and in some cases as advance meningitis and bacteremia (1). Resistance of Salmonella enteritidis to nalidixic acid and ceftriaxone is increasing (2). In the case of resistance to nalidixic acid, treatment would be quinolone (3). In endemic areas chloramphenicol would be used. The use of fluoroquinolones in children and pregnant women cause adverse events such as photosensivity, electrocardiographic abnormalities and tendinopathies (4). Bacteriophages are viruses that specifically bind to bacteria and they kill their host. Nowadays due to this specification and to prevent the spread of antibiotic resistance phenomenon the use of bacteriophage to control bacterial infections is taken into consideration (5-7). The benefits bacteriophage therapy compares to antibiotic therapy are: The frequency of these phages in the environment, High specification, The low cost of treatment and fewer side effects (8-10). For example, it is shown that prescribing specific phages against Klebsiella pneumoniae multi-drug resistant, bacteria was reduced the number of such bacteria in the mouse pneumonia (11). In the present study, we evaluated the therapeutic effects in specific phage against Salmonella enteritidis in animal models. 

## Methods

**Isolation and confirmation of bacteria**

Salmonella enteritidis was isolated from an outbreak in Tehran. Salmonella enteritidis using specific primers (table 1) designed for accurate diagnosis were confirmed by Multiplex PCR. Amplification was carried out in a thermocycler using the following cycling programme initial denaturation at 94°C for 5 min and 35 cycles of 30 s at 94°C, 30 s at 55–60°C and 2 min at 72°C, with a final extension for 5 min at 72°C [20]. After culturing on Hektoen enteric agar and bacterial antibiotic sensitivity testing in Luria Bertoni broth, was stored in the presence of 25% glycerol at -20 until the time of study was stored.

**Isolation, enrichment and purification of bacteriophage**

 For isolation specific bacteriophages wastewater samples were collected from the valiasar hospital in Tehran. Then the sample was placed in the refrigerator overnight to be deposited sediments. In summary, 15 ml of wastewater in 4500g was centrifuged for 10 min in 4 °C until bacteria cells were removed. The supernatant was passed through a filter 0.22µm. Filtered liquid added to a tube containing BHI broth in which was Salmonella enteritidis 1 x 106 cfu counts per ml. Then it was incubated overnight at 37° C shaking with120 RPM. The bacteriophage-host mixture was centrifuged at 5000g for 10 min and filtered through a 0.22µm-pore-size membrane filter. The filtrate liquid was subjected to the double layer method. This method was repeated three times until the isolation of single plaques. The bacteriophage was eluted from the final resulting plate by adding 5 ml of SM buffer on top of the plate and incubated at room temperature for 4 h with shaking. The supernatant was passed again through the filter and by using the double layer agar method; the presence of phage in filtered sample was evaluated. The suspension was passed through a filter 0.22µm. Phage solution was stored in 4 ° C until the time of the study.

**Morphology bacteriophage**

 Bacteriophage was studied in terms of size and morphology by using Transmission electron microscope. For this purpose, a copper grid coated with carbon was used which a drop of phage suspension was poured on it and then by using uranyl acetate 2% it was stained. Zeiss-Em10c- 80k transmission electron microscope was used.

**Animal model**

 In order to investigate the effect of a phage on Salmonella enteritidis, 42 Balb/c mice were prepared by the Department of Pharmacology. On average, the mouse's weight was 22-27g. We weighed them and divided into the six specific cages. Groups of mice were as follows: A: Bacteri (control) B: Bacteri+ bacteriophage (Simultaneous), C: Bacteri + bacteriophage Four days later, D: Bacteriophage + bacteri four days later E: Bacteri+ Ciprofloxacin (Simultaneous) F: Bacteri+ ciprofloxacin+ bacteriophage (Simultaneous). To place the mouse's in the stable state of stress, they were located in the room for 24 hours. For administration of Salmonella enteritidis, bacteria cultured on Hektoen enteric agar 24 hours before to bacteria stay in exponential phase. 0.5 McFarland (1.5×108cfu) suspension was prepared and ready to be administered to the animal. The suspension was prepared containing 2×109pfu bacteriophages for administration.2.5g Ciprofloxacin dissolved in 5 ml of distilled water and a uniform suspension was prepared for administration to the animal. 200µl of bacterial suspension in groups A, B, C, E, and F were treated using a gavage syringe. In Group B, to study the effects of Co administration of phage on bacteria in an animal model, 200µl of the phage suspension was given to each mouse. In group D, to study the efficacy of phage to prevent salmonellosis only 200µl phage was given on the first day.in group F, 200µl phage was administered. 160 µl ciprofloxacin was administered to groups E and F to study the effect on bacteria and synergism phenomenon) Three times in 24 hours). To study the efficiency of the phage in the treatment of salmonellosis , the phage was administered phage to group C in the fourth, seventh and tenth day 1 g of stool of an animal was taken and the results were evaluated. In each the specific day the mice weight was measured and the results were evaluated statistically. 

## Results

**Figure 1:**
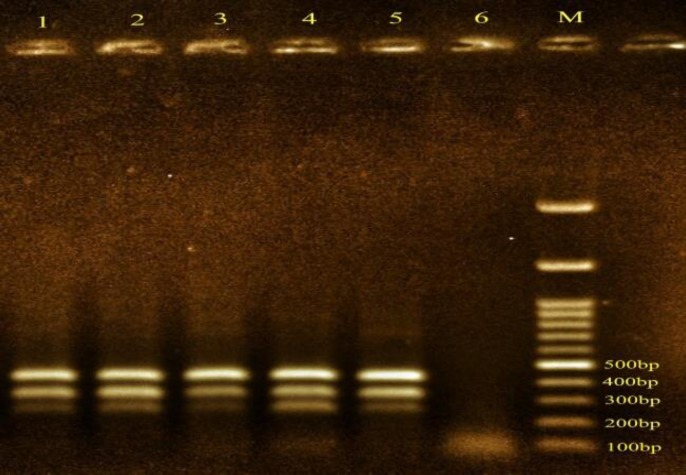
Detection of specific *Salmonella enteritidis *by multiplex PCR (Five samples were confirmed.), A: Marker- 100 bp

Salmonella enteritidis then isolated and cultured on Hektoen enteric agar medium and phenotypes were confirmed. Bacteria using specific primers was confirmed by Multiplex PCR. Five samples have three genes related to Salmonella Enteritidis (figure1).one sample was isolated for study. Susceptibility test showed that bacteria sensitive to ciprofloxacin by Kirby- Bauer disk. 

**Figure 2:**
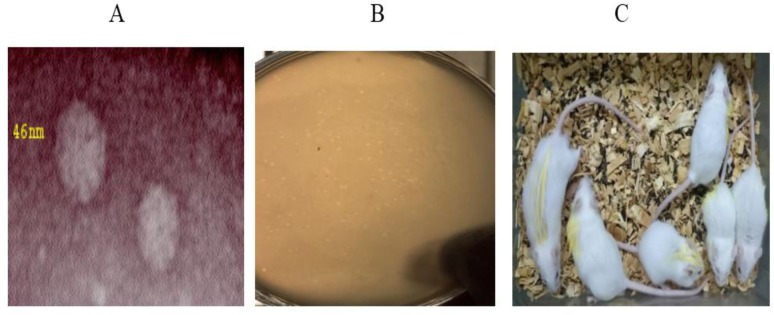
a:Purified plaques of bacteriophage b: : Pictures taken of electron microscope show the phage has a head size of 46 nm and without a tail c: To identify groups of mice, organs were highlighted in yellow. The difference of growth is evident in all groups. A: head B: loin C: Right hand D: left hand E: right leg F: left leg

 A lytic bacteriophages against Salmonella enteritidis isolated from samples of urban and hospital sewage and then it was purified.by using electron microscopy it is clear that, the phage is a virus particle with size of 46 nm with an icosahedral head and without a tail (figure 2).

**Figure 3:**
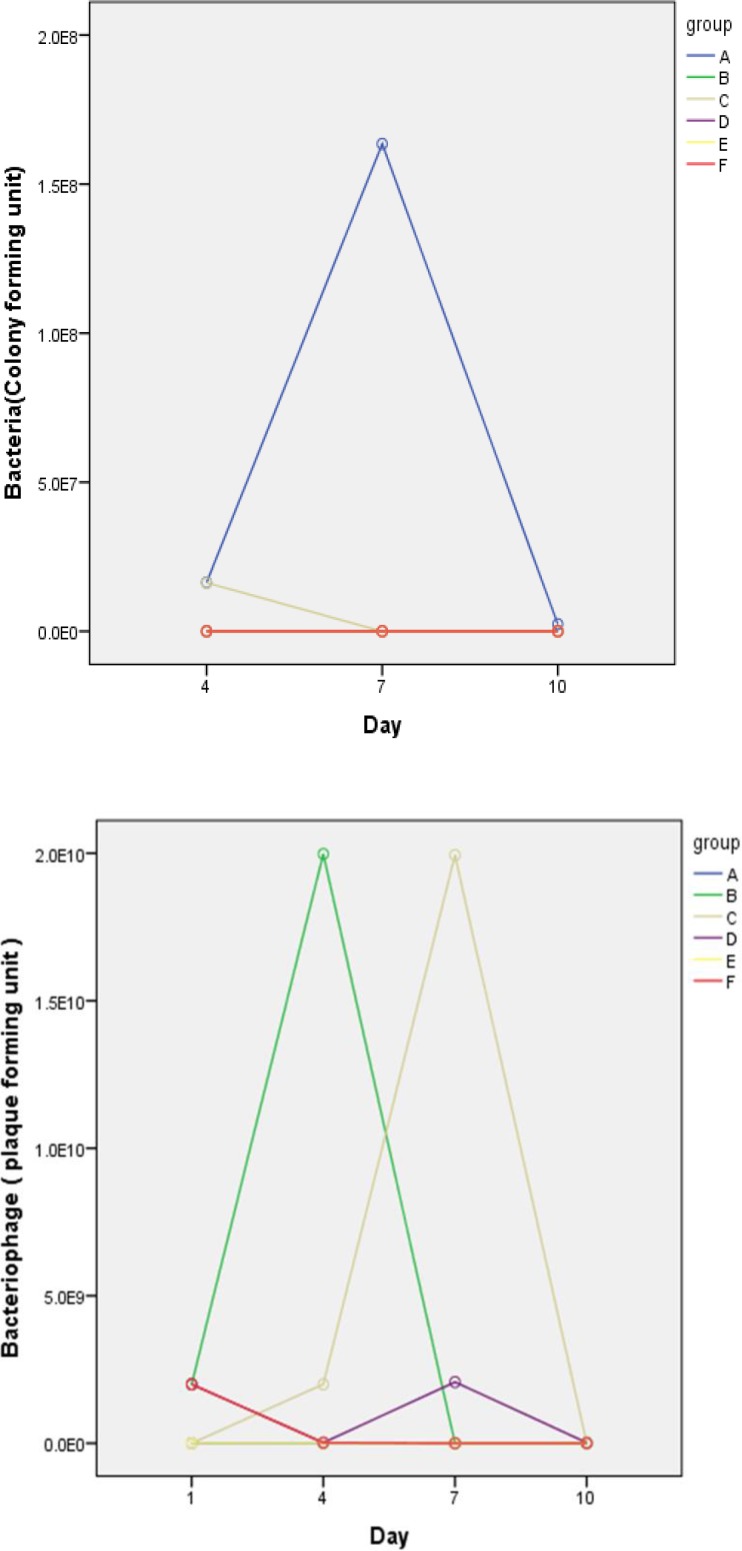
Profile plot diagram indicates changes in bacterial counts at three times in different groups. Group A is significantly different from the other groups. Due to the similarity of results in some groups (B,D,E,F), the curves overlap on diagram. Profile plot diagram indicates changes in bacteriophage numbers at four times in different groups. With the exception of Group D and F, the number of phage increased in the other groups, but the count fell on last days

In group A, the fourth, seventh and tenth days after the administration of bacteria, on average 1.6×107, 1.6×108, 2.3×106 cfu bacteria per gram of feces were counted. In this group of mice, after the administration of bacteria their activities decreased and their hair was ruffled and they lost weight. The stool was loose. At the end of the twelfth day, 5 mice died.in group B, in the fourth, seventh and tenth day Salmonella enteritidis was not isolated from stool samples. The number of disposal bacteriophages uptrend until the fourth day, but they decreased during the seventh day. Coadministrations inhibit the growth and proliferation of bacteria in the animal and no trace of salmonellosis were found. The activity and growth in this group was normal and hair was normal. In group C, The average on the fourth day 1.6×107cfu bacteria per gram stool was counted, but in the seventh and tenth days ,bacteria wasn’t isolated as a result of administration of bacteriophage. The results showed that the mice were treated with appropriate phage and only lactose-positive bacteria grew which wasn’t important to us. There was no death in this group.in group D, in the fourth, seventh and tenth day Salmonella enteritidis wasn’t isolated from stool samples (Table 2, figure 3). In this group, Activity and growth were normal and hair was normal. In group E, The bacteria were given to mice and after12 hours they were given ciprofloxacin every 8 hours. 

**Table 1:** Primers used for the detection of *Salmonella enteritidis *(20)

Primer Gene Sequence (5′–3′) Amplification product (bp)
ST11 Random sequence* CCAACCATTGCTAAATTGGCGCA 429 ST14 Random sequence GGTAGAAATTCCCAGCGGGTACTGG S1 *Spv*† GCCGTAGATACACGAGCTTA 250 S4 *Spv* ACCTACAGGGGCACAATAAC SEFA2 *sefA*‡ GCAGCGGTTACTATTGCAGC 310 SEFA4 *sefA* TGTGACAGGGACATTTAGCG

* Randomly cloned sequence specific for the genus Salmonella,

† *Salmonella* plasmid virulent gene,

‡ *Salmonella Enteritidis* fimbrial antigen gene.

**Table 2:** Average number of bacteria and phage is shown on different days in groups A-F

BC4 (cfu) BC7(cfu) BC10(cfu) BFC1(pfu) BFC4(pfu) BFC7(pf BFC10 (pfu)
A 1.6×10 1.6×10^8^ 2.3×10^6^ N NI NI NI B NG NG NG 2×10^9^ 1.9×10^10 ^ 2.8×10 2.7×10^4^ C 1.6×10^7^ NG NG N 2×10^9 ^ 1.9×10^10^ 2.8×10^5^ D NG NG NG 2×10^9^ 1.9×10 2×10^9^ 2×10^7^ E NG NG NG NI NI NI NI F NG NG NG 2×10^9^ 2×10^7^ 1.9×10^4^ 1.8×10^3^ Total 48 48 43 48 48 48 43

BC: bacteria count, BFC: bacteriophage count, NI: not isolated, NG: no grows, CFU: colony-forming unit, PFU: Plaque forming unit

**Table 3:** Average weight (grams) of mice is shown on different days (1,4,7,10) in groups A-F

group Weight(g) 1 Weight(g) 4 Weight(g) 7 Weight(g) 10
A 25.9 25 21.7 18.9 B 25.3 26.2 28.1 29.6 C 25.6 24.7 26.3 27.3 D 29.5 30.7 32.4 33.3 E 20.9 22.01 20.4 19.8 F 18.46 19.1 17.9 17.5 Total 48 48 48 48

**Figure 4:**
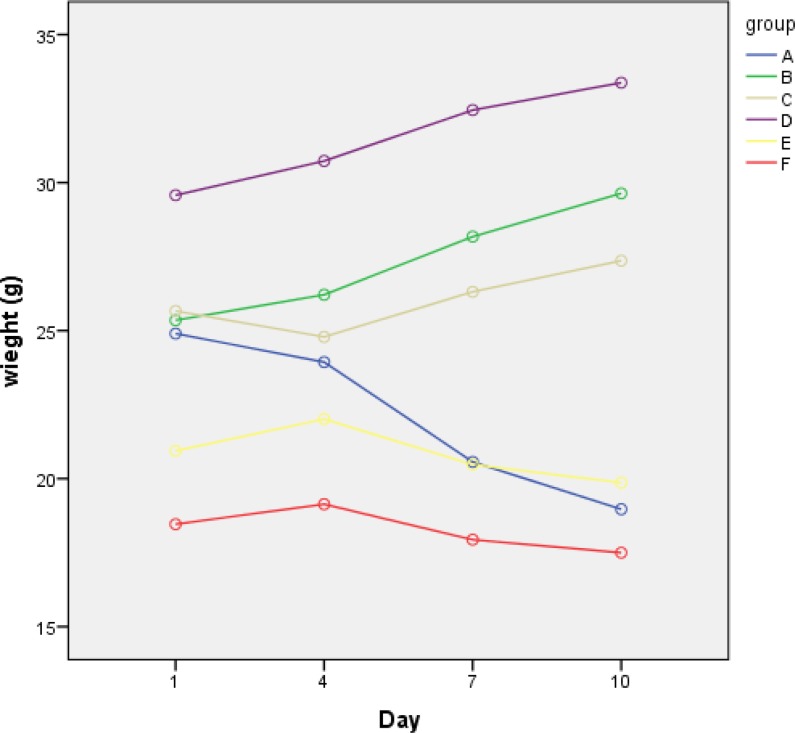
Profile plot diagram indicates changes of mice weight at four times in different groups. In groups where bacteriophage was use, body weight increased

Salmonella enteritidis and lactose positive bacteria were not isolated. Animal weight loss in this group was significant. In group F, the results were like group E, and weight loss was observed (Table 3, figure 4). To study changes of bacteria count during the time and intervention efficacy bacteriophage was used as analysis of variance with data Repeat. This indicates that the bacteria count changes during the time is meaningful. (P˂ 0.0001). To study changes of mice weight during the time and intervention efficacy bacteriophage was used as analysis of variance with data Repeat. This indicates that the mouse weight change during the time is meaningful. (p˂ 0.0001). 

## Discussion

Nowadays there are nearly 2,500 Salmonella serotypes and the most common species in worldwide is Salmonella enteritidis (12,13). Misusing of antibiotics in the treatment of diseases and over using them in the livestock industry has detected multiple drug resistance in Salmonella enteritidis. In recent years tendency to use bacteriophages to control pathogenic bacteria has increased (14). In this study, we isolated a bacteriophage that effectively controls salmonellosis caused by Salmonella enteritidis. In in vitro study it showed that isolated bacteriophage is specific for Salmonella enteritidis. The bacteriophage special plaque has not seen on Staphylococcus aureus, Escherichia coli, Yersinia enterocolitica, Salmonella typhimurium and Pseudomonas aeruginosa. This result is comparable to the findings of previous studies that specifically bacteriophage infects a species of bacteria (15). Despite of the reports that showed some bacteria can become resistant to bacteriophages via changing molecular receptor (16,17), But we did not see resistance during the twelve months. Hyun-wol Kang, identified 3wksl bacteriophage to control Salmonella enterica, serovar enteritidis and typhimurium in the food (18). Bourdin and colleagues have studied phage cocktail efficacy on E. coli that cause diarrhea (19). The results showed that phage cocktail 54-69% were effective on the bacteria. In this study, we demonstrate that oral administration of dose 2×109 pfu Salmonella enteritidis specific phage can prevent salmonellosis in mice. Profile plot diagram indicates changes in bacteria numbers during three times in different groups. Group A has significant difference with other groups. Profile plot diagram indicates changes in bacteriophage numbers during four times in different groups. With the exception of Group D and F, the number of phage increased in the other groups, but the counting fell last days. Profile plot diagram indicates changes of mice weight during four times in different groups. In groups which bacteriophage was used, the body weight increased. The results of this study showed that the isolated bacteriophage is able to control the disease. This phage has a very effective role to prevent infection caused by Salmonella enteritidis and the treatment of mice with salmonellosis. In Group E and F, no significant differences observed in the use of bacteriophage and antibiotics, but the use of antibiotics caused weight loss mice bodies. This phenomenon was not observed in the phage. This shows that the use of bacteriophage is much better than antibiotics the number of bacteriophage increased in the early days. Because it provided a host for the page, but by reducing the number of bacteria, the number of phages decreased last days.
